# 

**Published:** 1893-09

**Authors:** 


### CONTENTS:

Article I.—On Some Ways of Manipulating the Filling Materials. By Mr. Halliday. - - - - - - - 193

" II.—The Question of Local Anæsthetic Nostrums. By Edward C. Kirk, D. D. S. - - - - 206

" III.—Symptomatology of Some Dental Lesions. By F. W. Ryan, D. D. S. - - - - - 221

" IV.—Experiments with Amalgam. By B. - - - 227

" V.—Brieflets. By A. A. Burns, L. D. S. - - - 230

### EDITORIAL.

Development of Consumption. - - - - - - - 232

### MONTHLY SUMMARY.

Banding Roots. - - - - - - - - 233

Mutilation of the Teeth by Savages. - - - - - - 234

Root Treatment. - - - - - - - - 235

Unfermented Bread for Invalids. - - - - - - 236

Local Anæsthetic Nostrums. - - - - - - 238

Carcinoma of the Mouth. - - - - - - - 239

Dental Dots Distilled. - - - - - - - 240

